# ^99m^TcO_4_^−^ scintigraphic detection of follicular thyroid cancer and multiple metastatic lesions: A case report

**DOI:** 10.3892/ol.2013.1639

**Published:** 2013-10-23

**Authors:** CHANG-YIN WANG, BANG-RU XIAO, MEI-JUAN SHEN, YING SHEN, KUN-WEI CUI

**Affiliations:** 1 Department of Nuclear Medicine, Zhongnan Hospital of Wuhan University, Wuhan, Hubei 430071, P.R. China; 2 Department of Nuclear Medicine, Jingzhou Center Hospital, Tongji Medical College, Huazhong University of Science and Technology, Jingzhou, Hubei 434000, P.R. China

**Keywords:** follicular thyroid cancer, neoplasm metastasis, thyroid scan

## Abstract

^99m^TcO_4_^−^ thyroid imaging is often used to detect thyroid diseases that are confined to the neck. However, this examination is not frequently used to detect metastatic lesions of thyroid cancer in the whole body, while ^131^I imaging is often used to detect the metastases of differentiated thyroid cancers. The present study performed ^99m^TcO_4_^−^ thyroid imaging for a 69-year-old patient with a thyroid nodule and incidentally identified a lesion with abnormally increased ^99m^TcO_4_^−^ uptake in the chest of the patient. Furthermore, a whole-body scan was performed for this patient and multiple lesions with increased ^99m^TcO_4_^−^ uptake were identified. The results confirmed that these lesions were follicular thyroid cancer and the metastatic lesions were distributed in numerous locations. The results revealed that analysis of the whole body is significant when regional lesions with abnormally increased ^99m^TcO_4_^−^ uptake outside of thyroid tissues are identified by routine ^99m^TcO_4_^−^ thyroid imaging.

## Introduction

The incidence of follicular thyroid cancer is lower than that of papillary thyroid cancer in thyroid malignant tumors and is, therefore, the second most common thyroid malignancy (1). Follicular thyroid cancer is mainly characterized by a follicular structure. It is a differentiated thyroid cancer and demonstrates positive expression of the sodium-iodide symporter. Therefore, the tissues of follicular thyroid cancer generally uptake iodine, which is the biological basis for the detection of cancer lesions by ^131^I whole-body imaging and the treatment of follicular thyroid cancer by radioactive ^131^I (2,3). ^99m^TcO_4_^−^ and the iodide ion have a number of similar features, so ^99m^TcO_4_^−^ can also be absorbed by the thyorid (4). Follicular thyroid cancer may develop into regional nodal metastasis and may also progress into hematogenous metastasis (1). The accurate detection of recurrent and metastatic lesions of follicular thyroid cancer is significant in the staging of diseases and the evaluation of the therapeutic effect and prognosis (1). The present study describes a patient with follicular thyroid cancer whose metastatic lesions were detected using a ^99m^TcO_4_^−^ whole-body scan. Informed consent was obtained from the patient prior to the study.

## Case report

A 69-year-old female was admitted to Zhongnan Hospital of Wuhan University (Hubei, China) complaining of left stethalgia for three weeks. A physical examination revealed that the second rib in anterior left chest and the fourth thoracic vertebra were swollen and painful when palpated. The thyroid glands of the patient were intumescent and the left lobe to the isthmic portion was palpated as a hard and fixed nodule with an asperous surface, without pain. Laboratory examination demonstrated that the thyroid, liver and kidney functions, as well as the routine blood and urine test results, were all normal.

**Figure 1:**
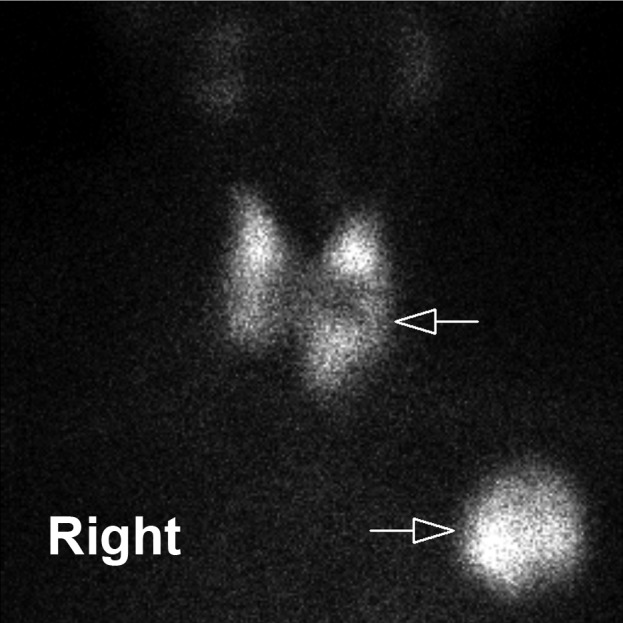
Thyroid static imaging with ^99m^TcO_4_^−^ showing a nodule in the middle portion of the left lobe of the thyroid with a decreased uptake and unclear edge, and an area of the left chest with a markedly increased uptake.

**Figure 2:**
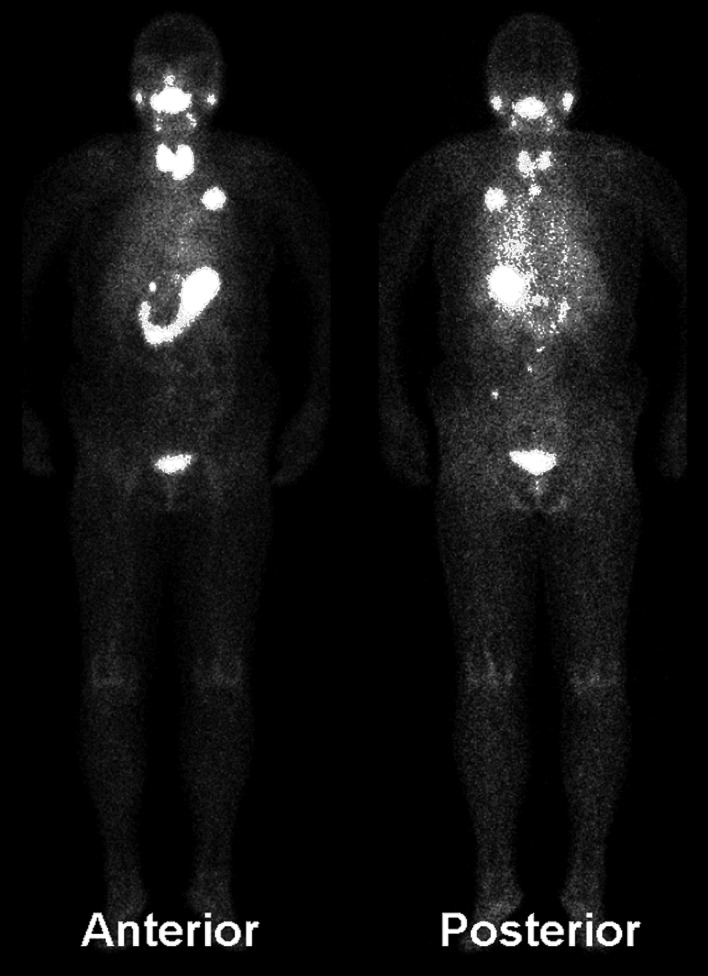
^99m^TcO_4_^−^ whole-body imaging showing one site of the left chest, two sites of thoracic vertebrae, two sites of lumbar vertebrae and one site of the left ilium with an increased uptake.

**Figure 3:**
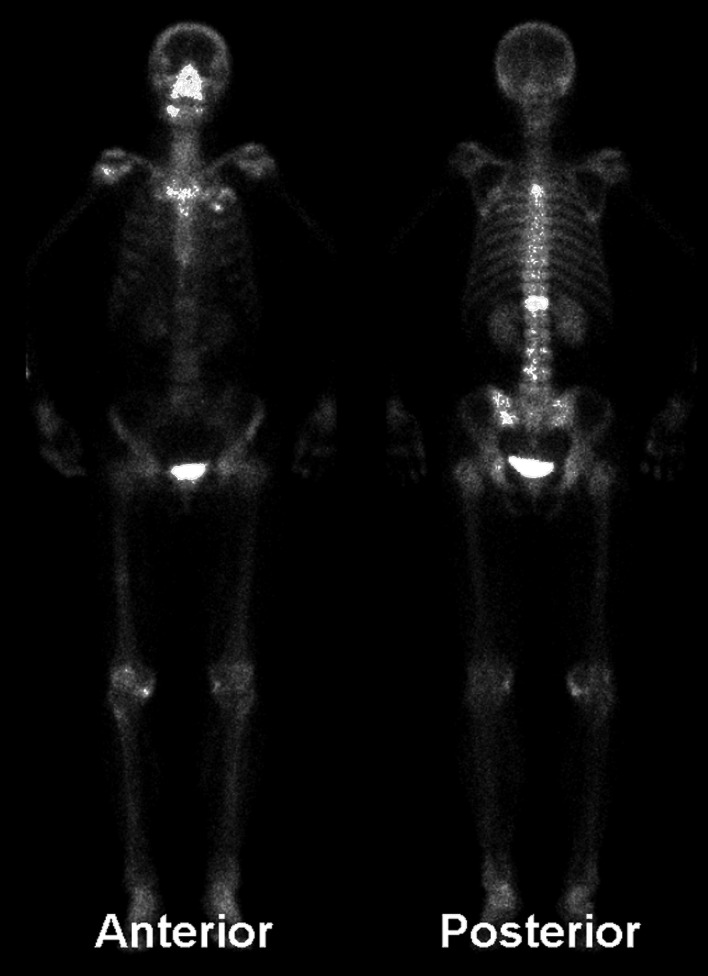
Whole-body bone imaging showing the left anterior branch of the 2nd rib, the 4th and 12th thoracic vertebrae and the midpiece of the right thigh bone with an increased uptake of ^99m^Tc-methylene diphosphonate. The 3rd and 4th lumbar vertebrae and the left posterior inferior iliac spine were suspected of abnormal uptake, whereas the partial osseous tissue of the 2nd rib presented with an osteolytic lesion.

Thyroid imaging was performed at 10 min following the intravenous injection of Na^99m^TcO_4_ at 185 MBq. The result demonstrated a regional area of markedly decreased ^99m^TcO_4_^−^ uptake with an irregular edge within the middle portion of the left lobe of the thyroid. A conglomerate area of increased ^99m^TcO_4_^−^ uptake was identified in the left chest (Fig. 1). Subsequently, ^99m^TcO_4_^−^ whole-body imaging was performed for the patient. The result revealed abnormal lesions of increased ^99m^TcO_4_^−^ uptake in the left chest, thoracic vertebrae, lumbar vertebrae and left ilium (Fig. 2). The following day, whole-body bone imaging was performed at 3 h following an intravenous injection of ^99m^Tc-methylene diphosphonate at 740 MBq. The imaging outcome demonstrated that the left anterior branch of the second rib, the fourth and twelfth thoracic vertebrae and the midpiece of the right thigh bone exhibited increased radioactive uptake. The third and fourth lumbar vertebrae and the left posterior inferior iliac spine were suspected of abnormal uptake, whereas the partial osseous tissue of the left anterior branch of the second rib demonstrated decreased radioactive uptake (Fig. 3).

**Figure 4:**
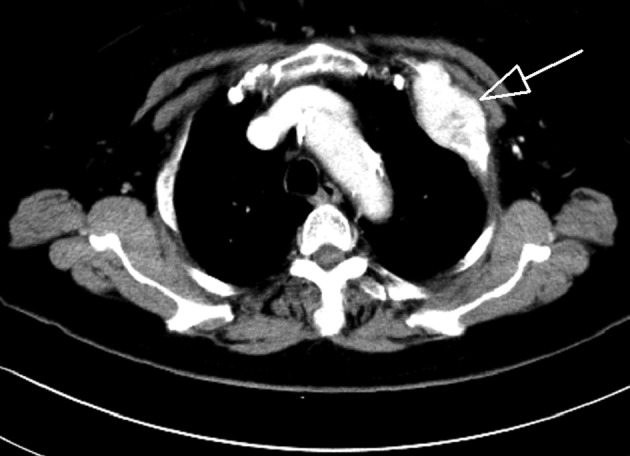
Computed tomography with contrast showing the 2nd rib with bony destruction and a markedly enhanced mass.

**Figure 5:**
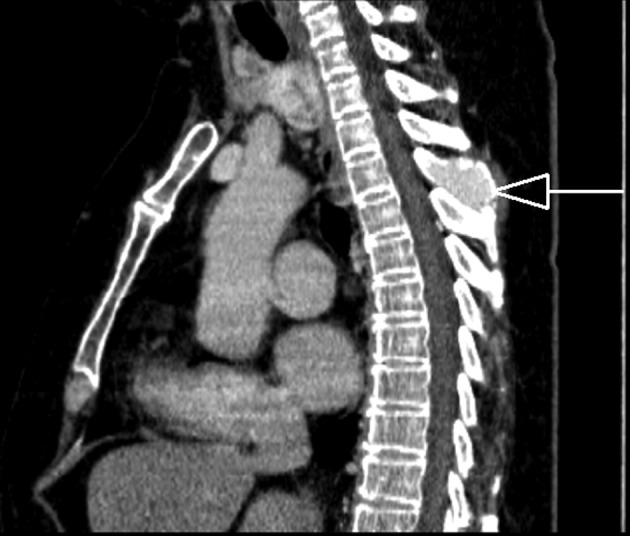
Computed tomography with contrast showing the 4th thoracic vertebra with bony destruction, and an enhanced lesion.

**Figure 6:**
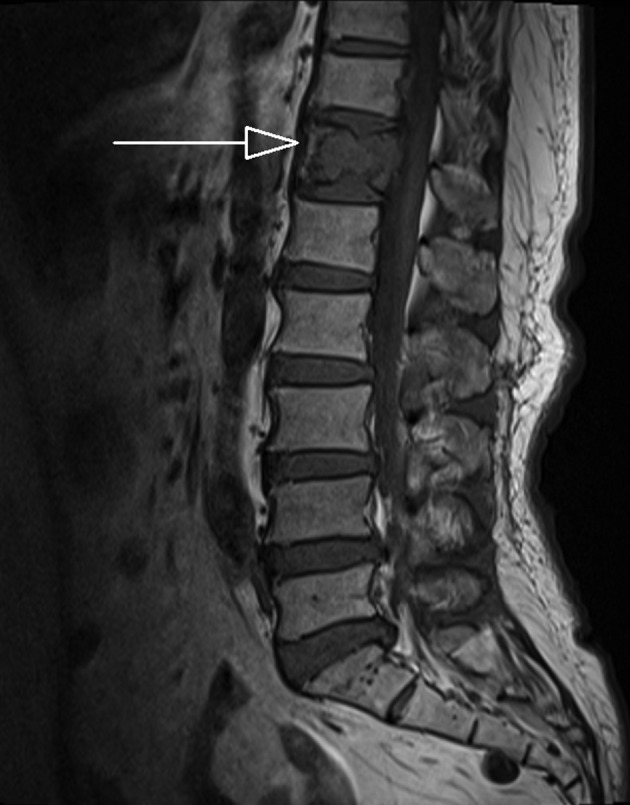
Magnetic resonance imaging showing the 12th thoracic vertebra with bony destruction.

**Figure 7:**
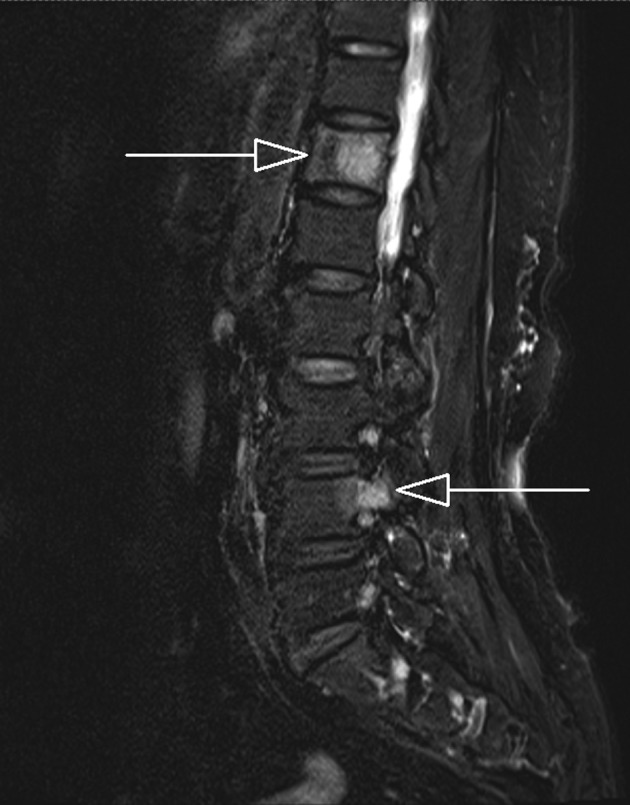
Magnetic resonance imaging showing the 12th thoracic vertebra and the pedicle of the 4th lumbar vertebral arch with an abnormal signal.

**Figure 8:**
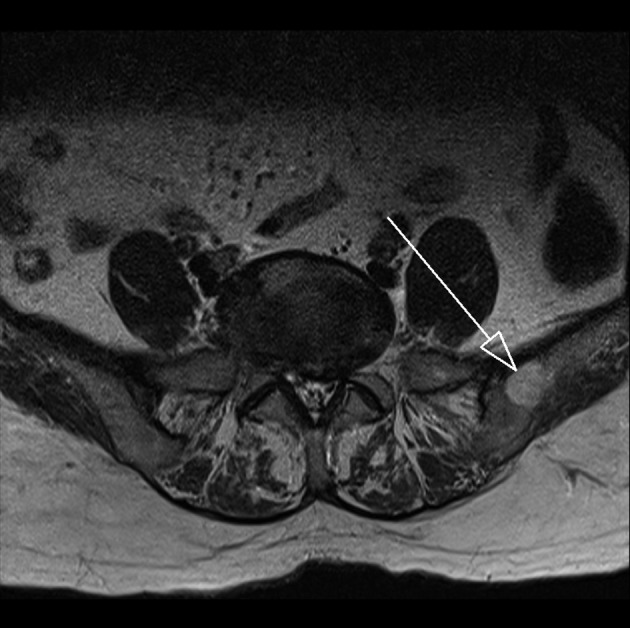
Magnetic resonance imaging showing the left ilium with an abnormal signal.

A computed tomography (CT) scan of the thoracic region revealed a mass on the left thoracic wall, bony destruction of the left anterior branch of the second rib (Fig. 4) and the destruction of the crest of the fourth thoracic vertebra (Fig. 5). A magnetic resonance imaging (MRI) scan of the vertebrae and pelvic cavity demonstrated that the twelfth thoracic vertebra (Figs. 6 and 7), the pedicle of the fourth lumbar vertebral arch (Fig. 7) and the left ilium presented an abnormal signal (Fig. 8).

The patient underwent a total thyroidectomy. The pathological results revealed that the left lobe nodule of thyroid was composed of follicular thyroid cancer tissue with peplos infiltration and tumor embolus formation of the small vessels.

## Discussion

^131^I whole-body imaging is often used for the identification of metastatic lesions of differentiated thyroid cancer following total thyroidectomy (2,3). When thyroid tissues are not completely resected, ^131^I is largely absorbed by the existing normal thyroid tissues (5). However, metastatic lesions universally have a low uptake of ^131^I and, therefore, the metastatic lesions are not displayed clearly (5). As a result, ^131^I whole-body imaging is not generally selected to identify the metastatic lesions of thyroid cancer in the presence of normal thyroid tissues. ^99m^TcO_4_^−^ is similar to the iodide ion to a certain extent (4); ^99m^TcO_4_^−^ and the iodide ion are mediated by the sodium-iodide symporter and are absorbed by thyroid follicular cells (4). Therefore, the two methods are often used in thyroid imaging (6). However, ^99m^TcO_4_ imaging is not generally used to detect the metastatic lesions of thyroid cancer in the presence of thyroid tissues and also following a total thyroidectomy (6–8).

Follicular thyroid cancer tissues are mainly composed of differentiated follicular cells. The proteins of the sodium-iodide symporter are predominantly distributed in the membrane of follicular epithelial cells, and cancer tissues with follicular cells express sodium-iodide symporter proteins (9,10), which are the pacing factors for which ^131^I and ^99m^TcO_4_^−^ are absorbed by the cancer tissues. The quantity of cancer tissues absorbing ^99m^TcO_4_^−^ correlates with the level of sodium-iodide symporter protein expression. Since cancer tissues are not well-differentiated, the level of sodium-iodide symporter protein expression is low (10,11). Accordingly, the lesions of thyroid cancer frequently manifest ‘cool nodules’ or ‘cold nodules’ of decreased ^99m^TcO_4_^−^ uptake (12). When thyroid tissues are not operated on, an increased ^99m^TcO_4_^−^ uptake of metastatic lesions of thyroid cancer is rare. Certain studies have reported an increased uptake of neck metastases of thyroid cancer in ^99m^TcO_4_^−^ thyroid imaging (7,8,13–15). However, to the best of our knowledge, there have been no studies with regard to the increased uptake of whole-body multiple metastatic lesions of thyroid cancer in ^99m^TcO_4_^−^ whole-body imaging. The present study incidentally identified a mass outside of the thyroid gland in a patient with thyroid ‘cool nodules’, which exhibited increased ^99m^TcO_4_^−^ uptake in the routine image field. Subsequently, a whole-body scan was performed. The result demonstrated that similar lesions of increased ^99m^TcO_4_^−^ uptake existed in multiple positions of the whole body. CT, MRI and radionuclide whole-body bone imaging confirmed that the multitudinous sites of increased uptake, which were detected by ^99m^TcO_4_^−^ imaging, contained tumor lesions. Therefore, despite the fact that ^99m^TcO_4_^−^ imaging is not routinely used to identify metastatic lesions of thyroid cancer, when regional lesions of increased ^99m^TcO_4_^−^ uptake are observed outside of the thyroid glands during routine field thyroid static imaging, further identification of the metastatic lesions of the whole body is significant.

Furthermore, ^99m^TcO_4_^−^ whole-body imaging has numerous advantages. The procedure is highly sensitive, as shown by the third and fourth lumbar vertebral lesions and the ilium lesion, which were detected by ^99m^TcO_4_^−^ imaging, but were not observed on the whole-body bone scan. MRI only identified the lesions of the fourth lumbar vertebra and the ilium, but not the third lumbar vertebral lesion. These results indicate that the sensitivity of ^99m^TcO_4_^−^ whole-body imaging is higher than that of MRI and whole-body bone scan at this time. Furthermore, the procedure is highly specific. The fact that ^99m^TcO_4_^−^ was able to be absorbed by thyroid cancer tissues under the mediation of sodium-iodide symporter proteins, confirms the diagnostic specificity for regional lesions. Abnormally increased ^99m^TcO_4_^−^ uptake of the lesions outside of the thyroid glands is a characteristic of metastatic thyroid cancer tissues (7,8,13–15), suggesting that the lesions likely originated from thyroid tissues. Finally, the procedure increases the quality of the diagnosis of the thyroid nodules. For the patient of the present study, the feature of the ‘cool nodule’ was not enough to discriminate between malignant lesions and benign tumors. However, the identification of the metastatic lesions extremely supported the diagnosis of the malignant thyroid nodule. The ^99m^TcO_4_^−^ imaging examination is able to scan the whole body of patients and, therefore, the detection area is extensive, which conduces to a complete detection of the lesions.
